# Associations between PTSD and pregnancy outcomes: systematic review and Meta- analysis

**DOI:** 10.1186/s12884-025-07545-9

**Published:** 2025-08-01

**Authors:** Natalie M. Zitoun, M. Karen Campbell, Joel Gagnier, Yasaman Mohammadi Kamalabadi, Facundo Garcia-Bournissen

**Affiliations:** 1 Department of Epidemiology & Biostatistics, University of Western Ontario, London, ON Canada; 2 Department of Pediatrics, Schulich School of Medicine & Dentistry, Western University, London, ON Canada; 3 Department of Obstetrics & Gynecology, Schulich School of Medicine & Dentistry, Western University, London, ON Canada; 4 Children’s Health Research Institute, London, ON Canada; 5 Lawson Health Research Institute, London, ON Canada; 6 Department of Surgery, Schulich School of Medicine & Dentistry, Western University, London, ON Canada

**Keywords:** Post-Traumatic stress disorder, Obstetrics, Pregnancy outcomes, Neonatal outcomes, Meta-analysis

## Abstract

**Importance:**

Post-Traumatic Stress Disorder (PTSD) is a prevalent and debilitating mental health condition. PTSD may be an important underlying mechanism in pregnancy and obstetric complications as well as adverse neonatal outcomes.

**Objective:**

The primary objective of this systematic review and meta-analysis is to evaluate associations between maternal PTSD and pregnancy, obstetric and neonatal outcomes.

**Data sources:**

A comprehensive literature search was conducted using Google Scholar, PubMed, and EMBASE databases. The search included recent data from various sources: databases, indexes, registries, abstracts, proceedings, references, experts, and institutions. Terms like PTSD, perinatal, pregnancy, and neonatal outcomes were used, the search was limited to English and human studies.

**Study selection:**

The inclusion criteria for detailed review focused on studies relevant to the topic, involving populations with perinatal PTSD, and assessing pregnancy, obstetric and neonatal outcomes. Two reviewers independently assessed the studies for eligibility, resolving discrepancies through discussion and consensus. Out of 369 initially identified studies, 40 met the selection criteria and were included in the review.

**Data extraction and synthesis:**

We followed PRISMA guidelines for data abstraction. Independent observers extracted data. Meta-analysis was conducted using a random-effects model, and evidence was evaluated according to GRADE guidelines. Statistical analysis was performed using R version 3.6.2.

**Results:**

40 studies were reviewed, including 27 prospective cohort, five retrospective cohort, four cross-sectional, and four case-control studies, totaling 157,708 pregnancies. Among them, 11,750 showed PTSD symptoms. Maternal PTSD was associated with smaller infant head circumference, sleeping/eating issues, reduced breastfeeding, and lower infant cortisol levels. Research varies on PTSD’s connection to low birthweight (LBW) and preterm birth (PTB). Meta-analyses of available data indicated significant associations: PTSD increased LBW risk (pooled OR 2.05; 95%CI: [1.27, 3.33]) and PTB risk (pooled OR 1.23; 95%CI: [1.11, 1.37]). GRADE analysis found overall low-quality evidence for LBW and PTB.

**Discussion & conclusion:**

PTSD in pregnancy links to adverse outcomes in both pregnancy and neonates. Preventing PTSD and addressing its causes during this period is vital for maternal, obstetric, and neonatal health. Further research, especially on pregnancy treatment effects, is necessary for informed clinical practices and policies.

**Supplementary Information:**

The online version contains supplementary material available at 10.1186/s12884-025-07545-9.

## Introduction

Posttraumatic stress disorder (PTSD) is a well-recognized chronic clinical disorder that most often occurs as a response to severe stressors or traumatic events and can persist for prolonged periods even after the stressor is gone. PTSD is a prevalent and typically debilitating psychiatric syndrome that can cause significant functional disturbances in patients’ lives [1]. To meet the current diagnostic criteria for PTSD, individuals must exhibit symptoms that have lasted for at least one month, causing distress or functional impairment in their daily activities. It is essential to rule out other potential causes, such as medication side effects, substance abuse, or comorbid medical conditions, to ensure an accurate diagnosis [2]. Women may experience PTSD during pregnancy either as a continuation of pre-existing PTSD from earlier life events or post-pregnancy as a result of trauma encountered during pregnancy, such as complications during labour, intimate partner violence, or other traumatic experiences related to the pregnancy itself [3]. Merging evidence suggests that PTSD may influence obstetric and neonatal outcomes through several potential mechanisms. Chronic stress associated with PTSD can lead to hormonal dysregulation, such as increased levels of cortisol and other stress-related hormones, which may adversely affect fetal growth and development. Additionally, PTSD has been linked to immune system dysregulation, which could contribute to inflammation and other complications during pregnancy. These biological mechanisms underscore the potential pathways through which PTSD may impact pregnancy outcomes, including low birthweight (LBW), preterm birth (PTB), and impaired neonatal development. Conversely, PTSD may also develop because of adverse obstetric or neonatal outcomes, creating a bidirectional relationship. Traumatic experiences such as preterm labor, emergency delivery, or neonatal complications can trigger PTSD in some women, highlighting the cyclical and complex nature of these interactions. Understanding this relationship is crucial for developing interventions that address both the causes and effects of PTSD in pregnancy.

There are inconsistent findings and limited reviews/meta-analyses on PTSD exposure in pregnancy and its association with adverse birth outcomes. Previous studies have reported mixed results regarding the associations between PTSD and key outcomes such as gestational age, birthweight, and mother-infant interaction. However, no comprehensive review has systematically synthesized this evidence while evaluating the quality of available studies. Our review aims to uncover PTSD’s association with pregnancy, obstetric, and neonatal outcomes, which is necessary for identifying at-risk groups and targeted interventions. By integrating findings from 40 studies, this review provides a robust synthesis of existing data while addressing key gaps in the literature. In using rigorous evaluation methods such as meta-analyses and the Grading of Recommendations Assessment, Development, and Evaluation (GRADE) guidelines, we aim to provide a reliable assessment of the evidence on this topic. The findings of this review will inform clinical practices, policy-making, and future research directions aimed at improving maternal and neonatal health outcomes.

## Methods

The protocol for this systematic review and meta-analysis is registered with the International Prospective Register of Systematic Reviews, PROSPERO (ID: (CRD42022358818). The Preferred Reporting Items for Systematic Reviews and Meta-Analyses (PRISMA) checklist was used to frame this systematic review and meta-analysis [4] (Appendix A, Table A1).

### Literature search

Studies were identified by searching the following databases: Google Scholar, PubMed, and EMBASE to identify relevant keywords in the title, abstract, and body of the articles.

### Inclusion & exclusion criteria

Studies reporting data on participants with PTSD during pregnancy and post-birth, compared to healthy pregnant participants without PTSD were included. The exposure in these studies had to be PTSD or related classifications (i.e Post Traumatic Stress Syndrome, trauma). For this manuscript, ‘PTSD exposure’ referred to either a formal diagnosis of PTSD or trauma exposure likely to result in PTSD symptoms. This broader criterion allowed for the inclusion of studies using varied definitions and tools for assessing trauma, such as self-reported questionnaires or clinical interviews. Pregnancy outcomes included low birthweight (LBW), preterm birth (PTB), gestational age (GA), breastfeeding, pre-eclampsia, gestational diabetes, mother-infant interaction, infant head circumference, development, temperament, and cortisol production. Low birthweight (LBW) was defined as < 2500 g, and included cases of LBW attributable to prematurity or cases of infants who are small for gestational age (SGA) presumably due to intrauterine growth restriction (IUGR). Studies using these specific definitions were included in the meta-analysis. Non-pregnant women studies without any PTSD-related exposure or trauma assessments other outcomes unrelated to pregnancy, obstetric, or neonatal outcomes were excluded from this review.

### Study selection

**Fig. 1:**
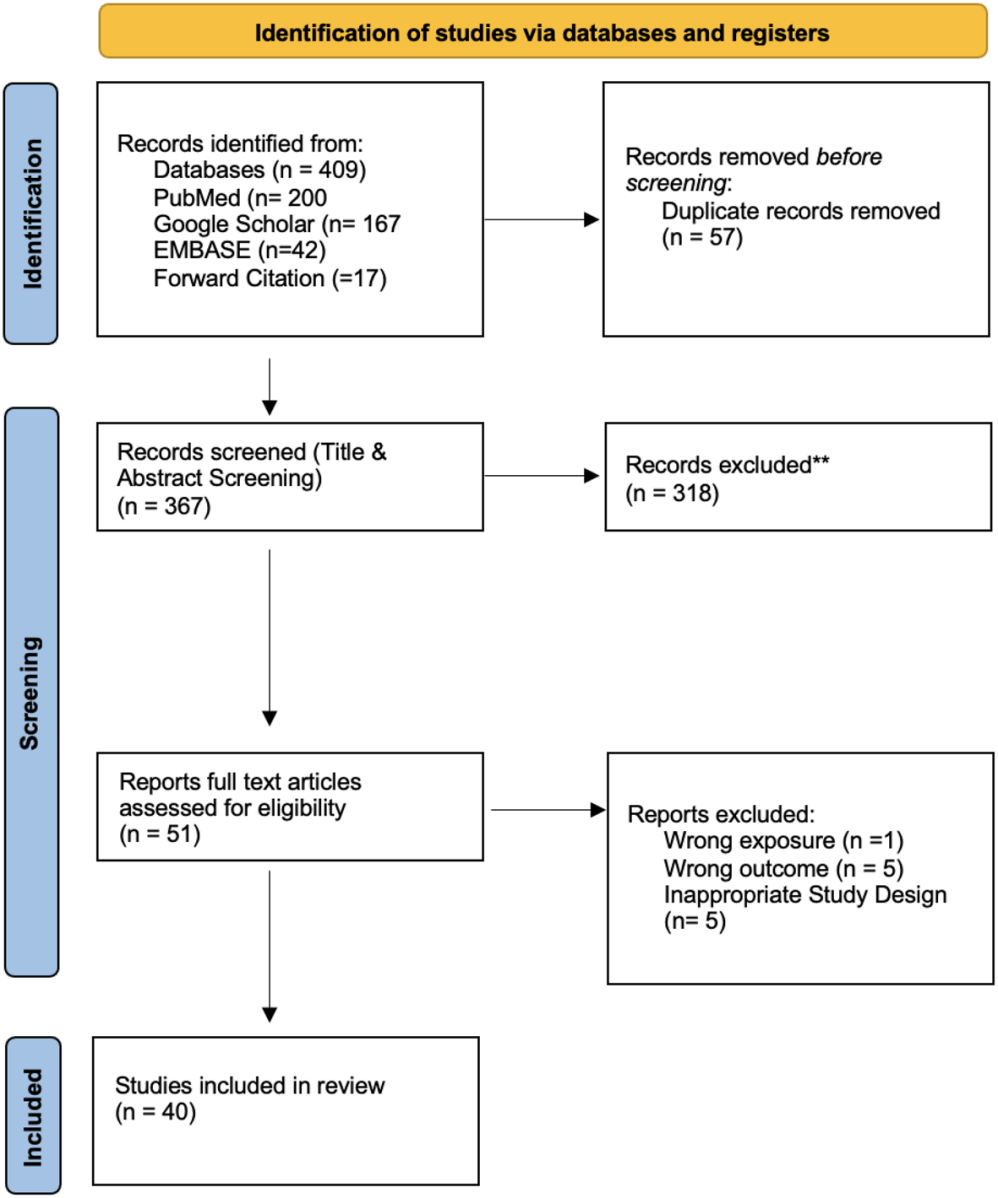
PRISMA Diagram of Study Identification and Selection

The initial search terms produced 409 articles: 200 from PubMed, 167 from Google Scholar and 42 from EMBASE, 57 duplicates were removed, and the 352 remaining articles were screened according to the eligibility criteria. Forward citation searching led to the identification of an additional 17 articles, resulting in a total of 369 articles (Appendix B). Two reviewers (NZ and YMK) screened articles in each database. Discrepancies between reviewers regarding study selection were resolved during the review stage. These articles went through title and abstract screening, which then resulted in 51 articles undergoing full-text review, 40 of the articles were found to be eligible for data extraction **(**Fig. 1**)**.

### Risk of bias assessment

The risk of bias (RoB) assessment was performed independently by two authors (NZ and YMK) using McMaster University’s “Tool to Assess Risk of Bias in Case-Control studies”, “Tool to Assess Risk of Bias in Cross-Sectional studies” and “Tool to Assess Risk of Bias in Case-Control studies” [5–7].

### Meta-analysis

The meta-analyses were conducted using statistical R version 3.6.2 using the “metafor” package [8]. Studies were included in the meta-analyses if point estimates of odds ratio were provided or if enough information was provided to be able to calculate odds ratios (OR). Odds ratios were chosen as the primary metric for this meta-analysis due to their widespread use in the included studies and their suitability for analyzing binary outcomes, such as LBW and PTB. While absolute effect sizes could provide additional granularity, the variability in reporting formats across studies made odds ratios the most consistent and interpretable choice for pooling results. A random effects model was used to estimate the pooled OR with their 95% confidence intervals (CI). Estimates from the most fully adjusted model were used. The heterogeneity between the study findings was assessed using the Cochran’s Q test at *p* < 0.05 and calculating I [2] values [9].

Adequate data allowed meta-analyses for LBW (< 2500 g, < 10th centile or < 2500 g, attributable to prematurity, small for gestational age (SGA), or intrauterine growth restriction (IUGR)), and PTB (< 37 weeks) Sensitivity analysis, using mixed-effects models, explored study factors’ impact on LBW and PTB outcomes. The aggregated Index, a composite measure that integrates multiple outcomes, such as low birthweight, preterm birth, and small for gestational age (SGA), into a single metric to capture a broader view of neonatal health outcomes was also calculated. R codes in Appendix E detail these analyses.

### Quality assessment: grading the evidence (GRADE)

The Grading of Recommendations Assessment, Development, and Evaluation (GRADE) system was applied to systematically assess the quality of evidence in the reviewed studies and was done using GRADE Pro GDT software (http://tech.cochrane.org/gradepro*)*, to determine the quality of the overall evidence for the two outcomes analyzed: LBW and PTB [10]. This assessment was performed independently by two authors (NZ and YMK). The GRADE approach evaluated study design, consistency, directness, precision, and publication bias to assess evidence quality.

## Results

### Overview of included studies in the review

A total of 40 studies, published between 2001 and 2022, were included in the review. These comprised 27 prospective cohort studies, five retrospective cohort studies, four cross-sectional studies, and four case-control studies. The studies involved participants from various countries, including the USA (*n* = 23), the United Kingdom (UK) (*n* = 7), Canada (*n* = 1), Brazil (*n* = 1), Peru (*n* = 1), South Africa (*n* = 2), Pakistan (*n* = 1), Israel (*n* = 1), Switzerland (*n* = 2), and Italy (*n* = 1). The analysis encompassed 157,708 pregnancies across these studies, with 11,750 pregnant participants having PTSD [11–49]. Notably, six papers did not report PTSD prevalence [12–17]. Participants’ mean age ranged between 23.3 and 33 years. Studies varied in PTSD assessment tools and birth outcome tools. Table 1 summarizes study designs, origin, age, PTSD cases, and statistical tests of all 40 studies.

**Table 1:** Summary table of studies (*n* = 40)

Study	Study design	Country	Age	Sample size	*N* (%) PTSD	PTSD tool assessment	Birth outcome measurement tool	Statistical analysis/ Testing method
Blackmore et al. [18]	Prospective Cohort	USA	Mean Age 24.42 (SD = 3.74)	358	139 (38.8%)	DSM	Obstetric outcomes were defined according to standard definitions and maternal and infant case notes reviewed by a maternal fetal specialist (E.K.*P*.)	Pearson’s χ2 analysis
Engel et al. [19]	Prospective Cohort	USA	Age Range: 18+	54	4 (7%)	PCL	Medical Records	Multivariable linear regression
Ferri et al. [20]	Prospective Cohort	Brazil	Age Range: 16–19	912	91 (10%)	CIDI	Hospital Interviews	Prevalence Ratios
Gelaye et al. [21]	Prospective Cohort	Peru	Mean Age: 27.9 years (SD = 6.1)	4408	1519 (34.4%)	PCL	Medical Records	beta coefficients (β) odds ratios (ORs) and 95% confidence intervals (CIs)
Koen et al. [22]	Prospective Cohort	outh Africa	Median = 29 years	544	108 (19.9%)	MINI	Medical Records	Regression Models
Lipkind et al. [23]	Case Control	USA	Age Range: = 18 - ≥ 35)	446	61 (12%)	PCL	Medical Records	multiple regression analysis
Maslow et al. [24]	Prospective Cohort	USA	Age Range: 15–49 years	3360	449(13.4%)	PCL	Medical Records	χ2 analysis, t tests, and generalized estimating equations (GEEs)
Morland et al. [25]	Prospective Cohort	USA	Mean Age = 27 Years	101	16 (16%)	PCL	A labor-and-delivery checklist, medical records	Descriptive and bivariate statistical analysis, including χ2 tests
Rashind et al. [26]	Case control	Pakistan	Age Range: = 20- ≥ 35)	450	84(18.7%)	MINI	Medical Records	Logistic regression analysis and univariate analysis
Rogal et al. [27]	Prospective Cohort	USA	Mean Age = 24.3 years (with PTSD) Mean Age = 24.5 (no PTSD)	1100	31 (3%)	MINI	Medical Records	χ2 test for two proportions
Rosen et al. [28]	Retrospective Cohort	USA	mean age = 25.9 years	148	38(25.7%)	University of Michigan Composite International Diagnostic Interview (UM-CIDI)	Medical Records	T tests and χ2 analysis
Seng et al. [29]	Prospective Cohort	USA	Unknown	839	98 (12%)	National Women’s Study PTSD Module	Medical Records	linear regression models
Weinreb et al. [31]	Case control	USA	Mean age = 26.3 Case Mean Age = 29.4 control	149	68(45.6%)	Four-item Primary Care-PTSD Screen	Medical Records	Propensity scores using logistic regression, Chi-square analyses, Repeated Measures Analyses of Variance (RM- ANOVA) and effect sizes
Xiong et al. [32]	Prospective Cohort	USA	Mean or range unknown (Range = 18 to ≥ 35)	277	13 (5%)	PCL	Medical Records	Chi-square tests and multiple logistic regression
Feeley et al. [33]	Cross-Sectional	Canada	Mean Age = 31	21	5 (24%)	PPQ	Medical Records	Descriptive statistics were computed for all variables, and Pearson product–moment correlations.
Harville et al. [34]	Prospective Cohort	USA	Age Range 18+	297	27(9%)	PCL	Medical Records	Bivariate and multivariable associations were examined using linear (for continuous) and logistic (for dichotomous) models
Haviland et al. [51]	Prospective Cohort	USA	Median = 33.2 years	787	157(20%)	Cohen’s 4-item Perceived Stress Scale	medical records	multiple imputation
Lutgendorf et al. [35]	Retrospective Cohort	USA	Age Range: = 17 - ≥ 35)	103,221	1657(2%)	ICD-9-CM code 309.81	medical records and ICD9CM code	Descriptive statistics and multivariable log-binomial regression
MacGinty et al. [36]	Prospective Cohort	South Africa	26 years	961	197(20%)	The Self-Reporting Questionnaire 20-item (SRQ-20)	Medical Records,	Linear regression models and multivariable models, Q-Q plot and Shapiro Wilk test, and VIF to check for multicollinearity
Yonkers et al. [50]	Prospective Cohort	USA	Mean Unknown (Range = ≤ 25 to ≥ 35 years)	2487	129 (5%)	Antenatal PTSD MPSS	Taken from self-report questionnaire and data from medical records	recursive partitioning, simple, and multivariable logistic regression analysis
Shaw et al. [38]	Retrospective Cohort	USA	Mean Unknown (Range = 19–40 + years)	16,334	30,149(19%)	ICD-9 diagnostic codes	Medical Records	unadjusted χ2 test bivariate analysis, and adjusted multivariate logistic regression
Parfitt et al. [39]	Prospective Cohort	UK	Mean Age = 33 years	45 dyads	Unknown	PDS	Parent-child interaction coded using CARE Index procedure	χ2 test Mann–Whitney
Parfitt & Ayers [40]	Retrospective Cohort	UK	Mean Age = 30 Years	151	8 (5%)	PDS	PBQ	Mann–Whitney U-tests, χ2 analyses, Spearman’s (r) rank order correlation test
Muller-Nix et al. [41]	Prospective Cohort (case-control?)	Switzerland	High preterm risk = 32 years Low preterm risk = 31 years Full term = 32 years	72 dyads (36 mothers, 36 infants)	High risk = 11(39%) Low risk = 4 (21% of mothers) Full term = 1 (4% of mothers)	Perinatal Posttraumatic Diagnostic Scale (PDS)	CARE Index procedure	Multivariate analysis of variance (MANOVA) Post-hoc test (Tukey). Item correlations regression analysis
yers et al. [11]	Cross Sectional	UK	Mean Age = 32 Years	64 families	3 (5%)	IES	Bethlehem Mother- Infant Interaction Scale	Wilcoxon signed ranks test, Spearman’s correlation. Multiple regression
Davies et al. [42]	Cross Sectional	UK	Fully Symptomatic (FS) = 26 years Partially Symptomatic (PS) = 30 years Non Symptomatic (NS) = 30 years	211	FT = 8 (3.8%) PS = 45 (21.3%)	PDSQ	MORS-SF ICQ MPAS	Scheffe ´ test, Descriptive statistics
Seng et al. [15]	Prospective Cohort	USA	Mean Age = 27 Years	566	43(7%)	(Perinatal PTSD) National Women’s Study PTSD Module	PBQ	Pearson R
Ionio et al. [43]	Prospective Cohort	Italy	Mean Age = 32.63 Years	58 dyads (29 mothers, 29 infants)	2 days postpartum = 2 (10.5% of mothers) 2 months postpartum = 4 (21.2% of mothers)	Perinatal Post Traumatic Stress Disorder Questionnaire (PPQ)	SFP coded using IRSS and MRSS	t-test and pearsons correlation
Parfit et al. [41]	Prospective Cohort	UK	Mean Age = 33 Years	75 dyads	Unknown	Perinatal Posttraumatic Diagnostic Scale (PDS)	ICQ PBQ	Spearman’s (rho) rank order correlation test. Paired-samples T-tests
Mcdonald et al. [44]	Cross-Sectional	UK	Mean Age = 32 years	81	14 (17%)	Postpartum PTSD) PTSDQ IES	MORS-SF PSI-SF	correlation and hierarchical multiple regression (HMRA). Spearman’s correlation coefficient
Beck et al. [45]	Cross-Sectional	USA	Unknown	903	Unknown	PSS-SR	Created questionnaire to abstract 23 new-onset physical problem after childbirth within first two months postpartum	Chi-square, Pearson product-moment correlation, t test, stepwise multiple regression, and hierarchical multiple regression analyses
Halperin et al. [46]	Prospective Cohort	Israel	Mean Age = 28.95 years	171	16 (9%)	PSS-SR	Child birth variables collected from self-report questionnaire 24–28 h after childbirth	series of t-tests, haierarchical linear regression in four steps. PTSD symptoms were used as a continuous variable.
Campbell et al. [47]	Prospective Cohort	USA	Age Range: 18+	445	19(4%)	PCL	Infant Behavior Questionnaire — Revised (IBQ-R)	Analysis of variance [ANOVA] with Bonferroni-adjusted *p* < 0.05 for all stress measures, WQS mixtures model
Bosquet Enlow et al. [48]	Prospective Cohort	USA	Mean Age = 27 Years	52 dyads	14 (27%)	PCL-C	IBQ-R ITSEA SFP-R	Mann–Whitney U tests SFP-R, mixed models and correlaiton
Parfitt et al. [39]	Prospective Cohort	UK	Unknown	42 families	Unknown	Birmingham Interview of Maternal Mental Health	ICQ PBQ	statistical Spearman’s (rho) rank order correlation test, mean ANOVA and multiple regression methods
Pierrehumbert et al. [14]	Prospective Cohort	Switzerland	Parent of high risk of preterm infant = 31 years Parent of low risk of preterm infant = 30 years Control = 32 years	75 families	Unknown	PPQ	SCL	r correlation coefficients and t tests
Yehuda et al. [16]	Prospective Cohort	USA	Unknown	38 dyads	Unknown	PCL	level of cortisol	Pearson’s correlational analyses, F test
Nillni et al. [17]	Prospective Cohort	USA	Mean Age = 298 Years	318	Unknown	Primary Care PTSD Screen for DSM-5 (PC-PTSD)	Medical records	logistic regressions, one linear regression and Spearman’s rho correlations
Seng et al. [30]*	Cross-Sectional	USA	Mean Age = 23.3 years (PTSD) 24.0 years (comparison)	1093	455 (42%)	(Antenatal PTSD) ICD-9 code taken from clinical records	Rates of hospital coding for obstetric complications	logistic regression
Shaw et al. [49]	Retrospective Cohort	USA	19–48+	4408	1519(34%)	ICD-9 diagnostic codes	medical records	multivariable-modified Poisson regression

Abbreviations: Diagnostic and Statistical Manual of Mental Disorders (DSM), Post-traumatic Stress Disorder Checklist-(PCL), Perinatal Posttraumatic Diagnostic Scale (PDS), Impact of Event Scale (IES), Perinatal Post Traumatic Stress Disorder Questionnaire (PPQ), Postpartum PTSD questionnaire scores (PTSDQ), Mini International Neuropsychiatric Interview (MINI), PTSD Scale-Self Report for DSM-5 (PSS-SR), Composite International Diagnostic Interview (CIDI), The Self-Reporting Questionnaire 20-item (SRQ-20), Primary Care PTSD Screen for DSM-5 (PC-PTSD), International Diagnostic Code Descriptions (ICD-9)

### GRADE assessment

The GRADE assessment evaluated study design, consistency, directness, precision, and publication bias. Studies were graded down for factors such as risk of bias, inconsistency, indirectness, imprecision, and publication bias. Conversely, studies were graded up for strong effect sizes, or if all plausible biases would reduce the apparent effect. Sensitivity analyses and risk of bias assessments were integrated to address limitations and variability in study quality. GRADE assessment found low-quality evidence for birthweight and PTB studies. LBW and PTB outcomes were compromised by bias due to intermediate risk of bias, indirectness in PTSD assessment tools and pregnant populations, and imprecision (wide confidence intervals). LBW outcome had validity issues due to high heterogeneity (> 70%). Results showed that PTSD increased the odds of LBW (OR = 2.05; 95% CI 1.27–3.33) in 11,798 participants and increased the odds of PTB occurrence (OR = 1.23; 95% CI 1.11–1.37) in 128,533 participants (Table 2).

**Table 2:**
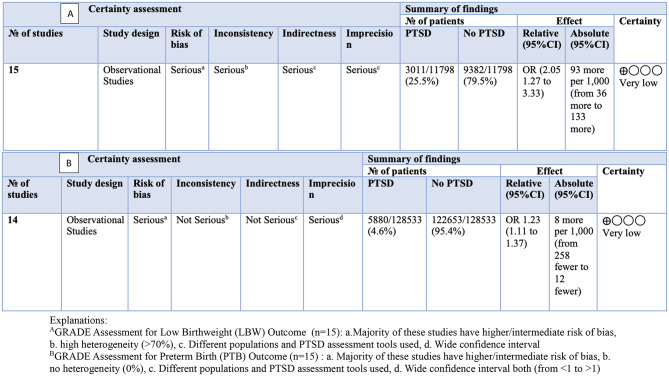
GRADE assessments for low birthweight (LBW) and preterm birth (PTB) outcomes

### Risk of Bias

**Fig. 2:**
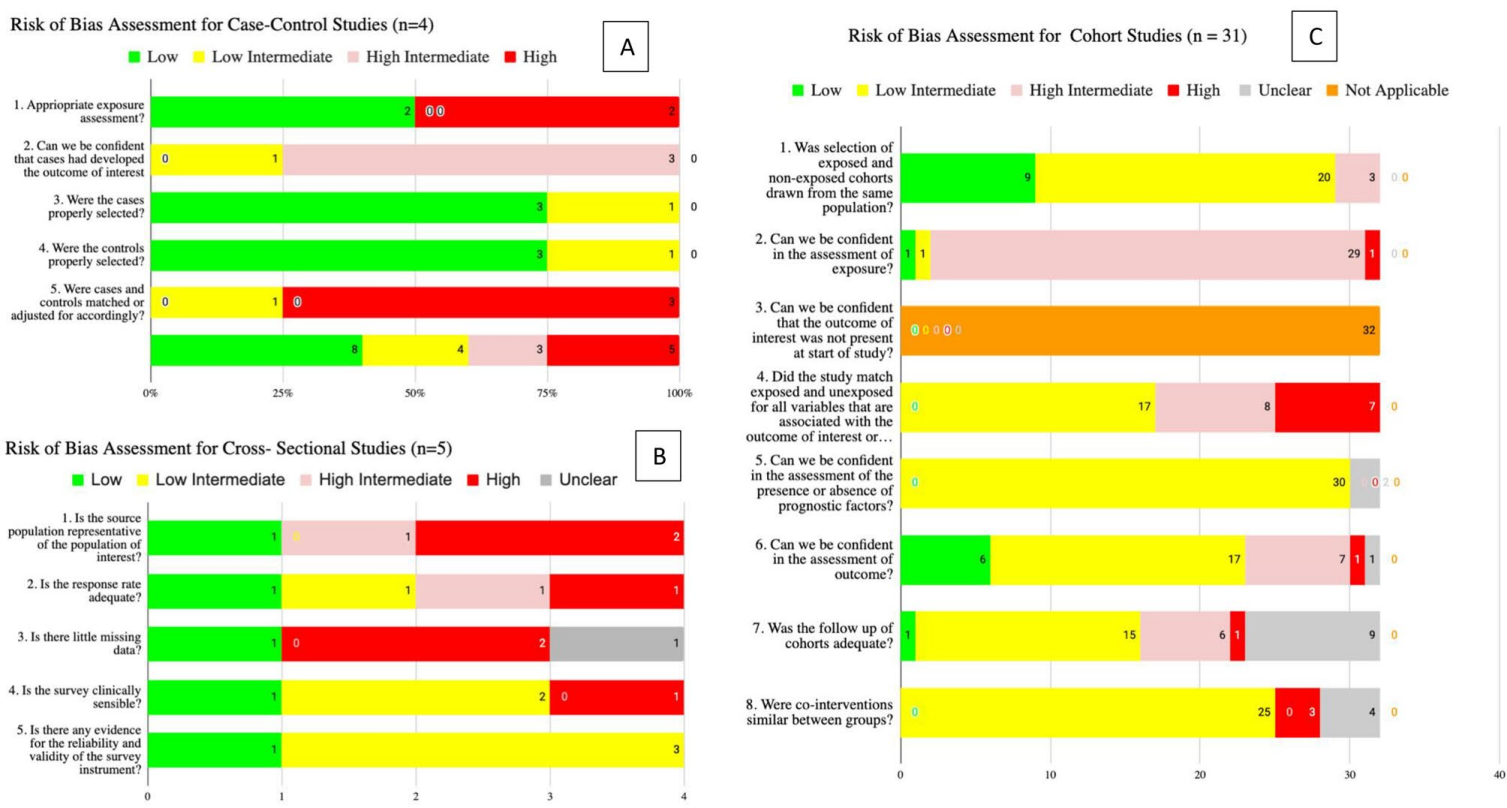
Risk of Bias (ROB) Assessments for all included studies. Note: Panel **A** refers to risk of bias assessment for case-control studies (n=4). Panel **B** refers to Risk of Bias Assessment for Cross-Sectional Studies (n=5). Panel **C** refers to Risk of Bias assessment for Cohort Studies (n=31)

Figure 2. shows risk of bias (RoB) finding. For the case-control studies, none had a certain low RoB. 75% (*n* = 3) didn’t match or adjust for prognostic variables. For the 5 cross-sectional studies, all lacked a representative source population. The Ayers et al. (2007) study had overall high RoB [11]. For the 31 cohort studies. 91% (*n* = 29) showed higher intermediate RoB in assessing PTSD due to recall bias and self-reported measures. However, these studies generally demonstrated low intermediate RoB in several other key areas including; selecting exposed/unexposed cohorts from the same populations (63%, *n* = 20), assessing prognostic factors (94%, *n* = 30), and similar co-interventions between groups (78%, *n* = 25).

### Study characteristics

Studied infant outcomes included LBW, PTB, gestational age, mother-infant interaction, development, cognition, negative affectivity, head circumference, temperament, breastfeeding duration, and sleep/eating patterns. Obstetric complications Obstetric complications were defined as medical conditions or events occurring during pregnancy, labor, or delivery that can impact maternal or neonatal health. Examples include gestational diabetes, preeclampsia, prolonged labor, placental abruption, and other similar conditions. Studies assessed multiple infant outcomes. Commonly studied were infant LBW (*n* = 15), PTB (*n* = 14), gestational age (*n* = 9), and mother-infant interaction (*n* = 10). Fewer looked at obstetric complications (*n* = 5), head circumference (*n* = 3), infant temperament (*n* = 2), negative affectivity (*n* = 2), breastfeeding (*n* = 2), infant development (*n* = 1), cognition (*n* = 3), and sleep/eating patterns (*n* = 1) [11–49].

### Low birthweight (LBW)

The study characteristics and main findings related to each outcome category are provided in Appendix C. A total of seven studies found that maternal PTSD was significantly associated with LBW (*n* = 7) [20, 23, 24, 26, 28, 29, 32, 33]. Contrary to these seven studies, eight (*n* = 8) studies found no significant association between maternal PTSD and low infant birth weight **(Table C1)** [18, 19, 21, 22, 25, 27, 31, 32].

### Gestational age (GA)

Table C2 presents the findings for all nine studies exploring the association between PTSD exposure during pregnancy and gestational age at birth (*n* = 9). One study found that maternal PTSD was significantly associated with lower gestational age [34]. The remaining eight (*n* = 8) studies found no significant association between antenatal PTSD and gestational age [18, 19, 21, 22, 27, 31, 35].

### Preterm birth (PTB)

Table C3 presents the findings for all 14 studies between PTSD during pregnancy and infant PTB. Seven of these studies were among the nine studies assessing GA as a continuous variable [21–23, 27, 31, 34, 35]. Six out of these fourteen studies (*n* = 6) found that maternal PTSD was significantly associated with PTB [21–23, 34, 38, 50]. The remaining eight studies (*n* = 8) found no significant association between antenatal PTSD and PTB [25, 27, 29, 31, 32, 35, 36, 51].

### Mother-infant interaction

Twelve studies (*n* = 12) reported findings between perinatal PTSD and mother-infant interaction, and are presented in **Table C4.** For two of these studies (*n* = 2), breastfeeding duration was included as a sub-category of mother-infant interaction. Eight of these twelve studies (*n* = 8) found that maternal PTSD was significantly associated with hindered mother-infant interaction [15, 33, 40–43, 45, 46].

### Mother-infant interaction: breastfeeding duration

When looking at breastfeeding as a sub-category of mother-infant interaction, there was some evidence for an association between PTSD exposure during pregnancy and reduced breastfeeding in infants (*n* = 2) [45, 46]. **(Table C4)**.

### Infant & neonatal complications

Nine studies (*n* = 9) explored perinatal PTSD’s link to neonatal complications, summarized in Table C5. Infant issues studied: Infant negative affectivity (*n* = 2); Infant temperament (*n* = 2); Infant Cognition & Development (*n* = 4); Infant Sleeping/Eating Behavior (*n* = 1); Lower Infant Cortisol levels (*n* = 1). Significant associations appeared between maternal PTSD and infant/neonatal problems in five of nine studies (*n* = 5) [12, 14, 16, 42, 47]. When looking at sleeping/eating behaviour, maternal PTSD was significantly associated with sleeping and eating difficulties in premature infants. There was also evidence that maternal PTSD was associated with lower infant cortisol levels [14, 16].

### Neonatal head circumference

Three studies explored an association between PTSD exposure during pregnancy and neonatal head circumference. As presented in **Table C6**, all three studies found some degree of association between antenatal PTSD or symptoms of antenatal PTSD and reduced infant head circumference [19, 22, 36].

### Obstetric complications

The findings between PTSD exposure during pregnancy and obstetric complications (*n* = 5) are presented in **Table C7**. Three of these five studies found significant associations of maternal PTSD with obstetric complications [17, 30, 49].

### Overall associations

Overall, the evidence in the papers reviewed consistently supported significant associations between maternal PTSD with infant sleeping & eating difficulties [aggregated index = 0.31; *p* < 0.01], lower infant salivary cortisol levels [F = 8.0, df = 1, 29; *P* = 0.008], reduced breastfeeding, and reduced infant head circumference (Table C8).

### Meta-Analysis and heterogeneity

**Fig. 3:**
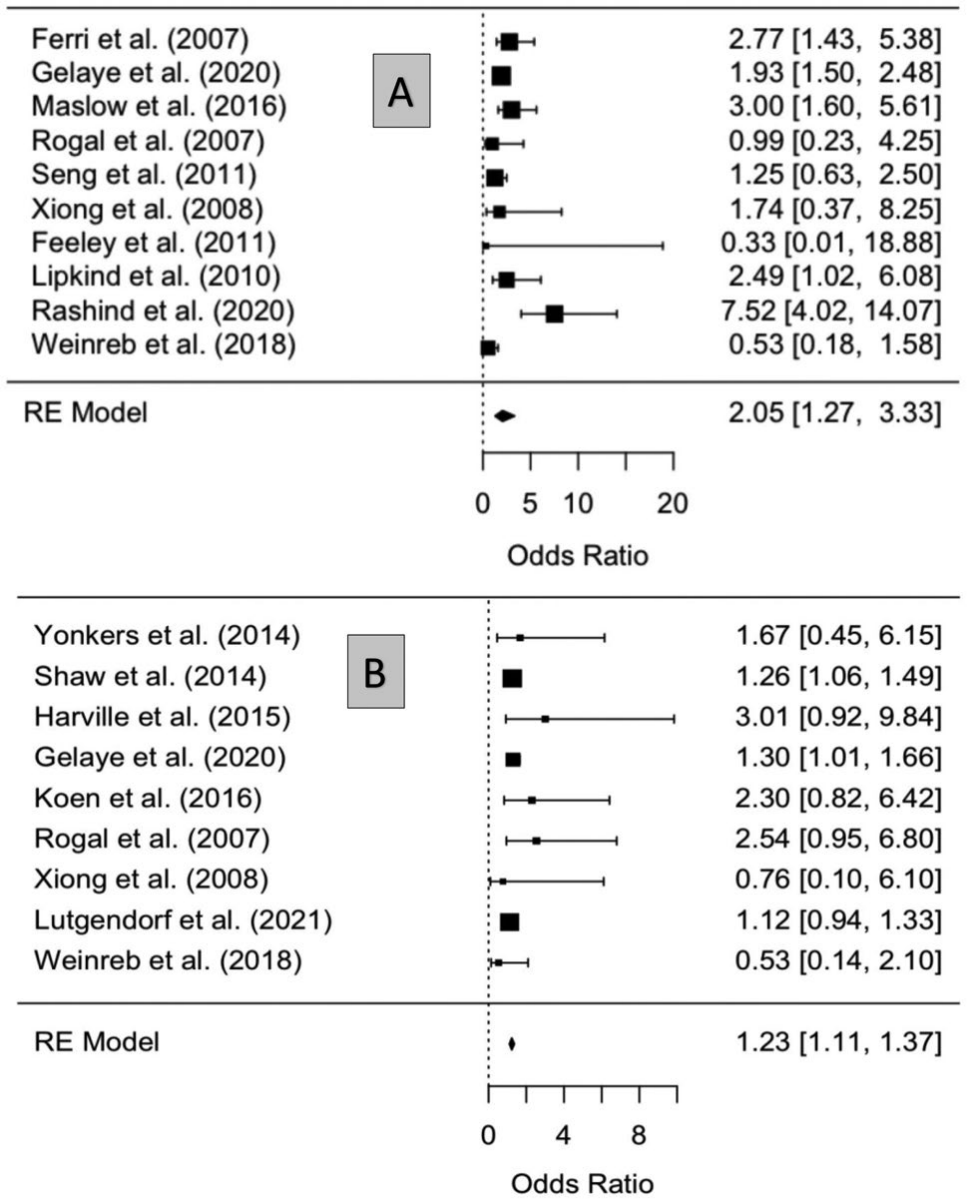
Statistical summary and forest plot of for the association between PTSD exposure with LBW and preterm birth. Note: Panel **A** refers to Birthweight Forrest Plot: Statistical summary and forest plot of for the association between perinatal PTSD and infant birthweight: [(P = 0.0035), (n=10)]. Panel **B** refers to Preterm Forrest Plot: Statistical summary and forest plot of OR for the association between perinatal PTSD and preterm birth [P= 0.0002, (n=9)]

A mixed-effects meta-analysis revealed significant associations between perinatal PTSD exposure and low birthweight (LBW) (pooled OR 2.05; 95% CI [1.27, 3.33], Ι² = 74.54%) across 10 studies with high heterogeneity. Similarly, PTSD exposure was associated with preterm birth (PTB) (pooled OR 1.23; 95% CI [1.11, 1.37], Ι² = 0%) across 9 studies with low heterogeneity (Fig. 3).

Sensitivity analyses further explored these outcomes. For LBW, the analysis revealed no significant residual heterogeneity (QE [df = 1] = 0.4065, *p* = 0.5237), but moderator tests indicated significant heterogeneity among moderators (QM [df = 8] = 28.0447, *p* < 0.05). Further investigation identified a single study [26]as a significant outlier (*p* = 0.0021). Study design also influenced LBW results, with significant effects observed in case-control studies (*p* = 0.0315) and prospective cohort studies (*p* = 0.0086).

**Fig. 4:**
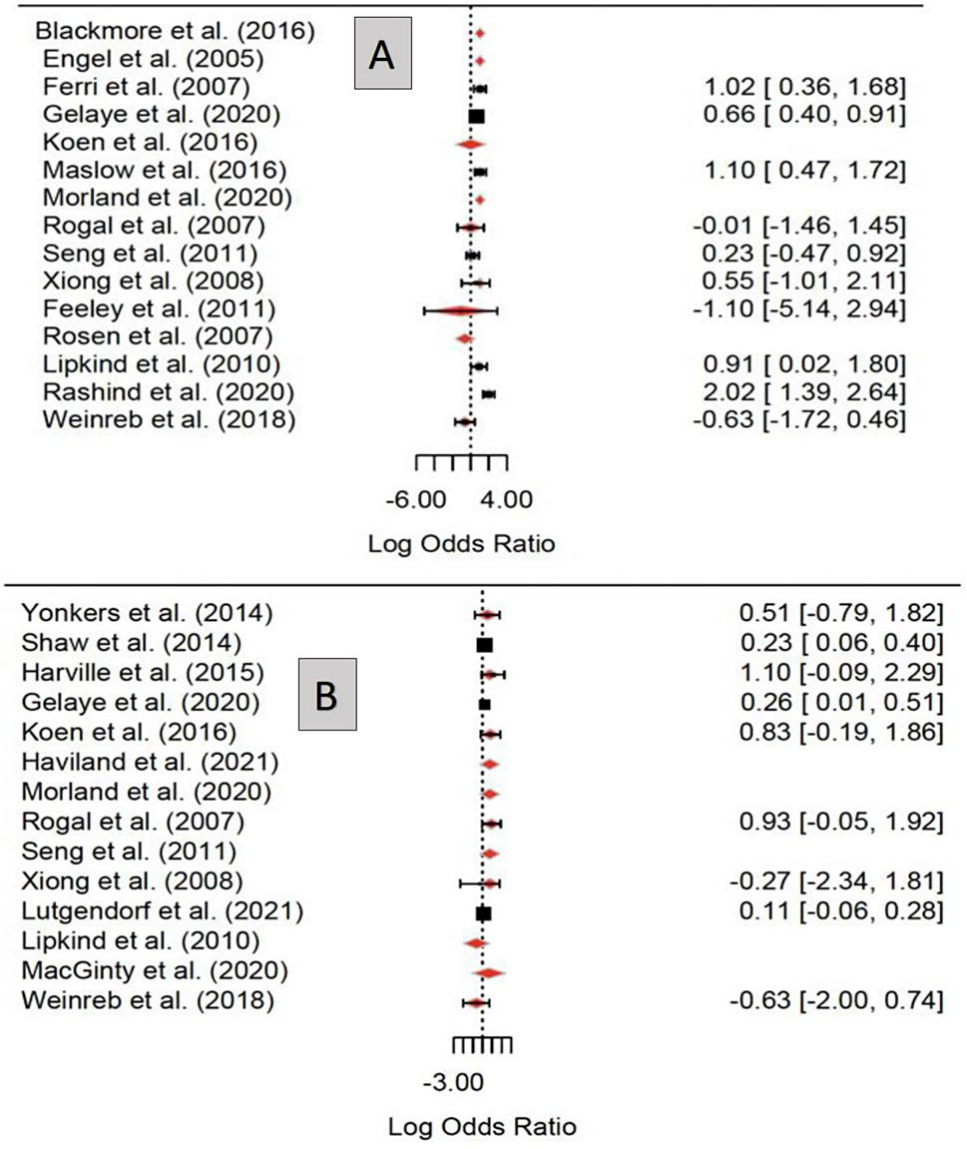
Sensitivity Analyses for the association between PTSD exposure with LBW and preterm birth. Note: Panel **A** refers to BWT Sensitivity Analysis Forest Plot (n=15). Panel **B** refers to PTB Sensitivity Analysis Forest Plot (n=14)

For PTB, sensitivity analyses found no significant residual heterogeneity (*p* = 0.4858), and moderator tests were not significant (*p* = 0.2570). These findings are illustrated in Fig. 4 and detailed in Appendix D.

## Discussion

### Summary of overall findings

Our review represents the most comprehensive synthesis to date on the relationship between maternal PTSD and pregnancy, obstetric, and neonatal outcomes, incorporating findings from 40 studies. Compared to Cook et al. (2018), which analyzed 21 studies, our review extends the scope by evaluating a wider range of outcomes, including infant birthweight, preterm birth, gestational age, mother-infant interaction, breastfeeding, cognitive development, temperament, and obstetric complications. While Cook et al. focused on fetal growth and gestational duration, our findings demonstrate broader implications of maternal PTSD, highlighting significant associations with low birthweight (LBW), preterm birth (PTB), diminished mother-infant interaction, and neonatal complications [52].

### Detailed discussion of key findings

Preterm birth (PTB) and low birthweight (LBW) were among the most significant findings in our review. Six out of 14 studies (43%) identified an association between maternal PTSD and PTB, with our meta-analysis of nine studies (64%) confirming increased odds of PTB for mothers with PTSD exposure. These findings align with Sanjuan et al. (2021), who reported similar associations [37]. However, variations in PTB definitions across studies and the inclusion of only nine studies in the meta-analysis present limitations. GRADE analysis further indicated low-quality evidence, suggesting caution in interpreting these results. Similarly, LBW was linked to maternal PTSD in seven out of 15 studies (47%), with meta-analysis of 10 studies (67%) revealing PTSD-exposed mothers had over twice the odds of delivering LBW infants. Most studies report LBW prevalence below 10% in normal pregnancies, allowing odds ratios to approximate risk ratios [53]. While sensitivity analysis showed no significant heterogeneity, GRADE assessments highlighted uncertainty due to methodological limitations and potential bias. Diminished mother-infant interaction emerged as a key concern, with 67% of studies demonstrating an association between perinatal PTSD and reduced bonding, particularly shorter breastfeeding durations. Breastfeeding is a critical aspect of infant health, with its disruption linked to adverse health outcomes [54]. Preliminary evidence also highlighted associations between maternal PTSD and smaller infant head circumference (3/3 studies), which is linked to developmental delays, intellectual disability, and neurological risks [55]. These findings emphasize the need for targeted interventions to support mother-infant bonding in PTSD-affected populations. Evidence on neonatal and infant behavioral outcomes was less consistent. Infant negative affectivity (NA) and difficult temperament, linked to later-life challenges such as anxiety and somatic disorders, were associated with maternal PTSD in one out of two studies [42]. Similarly, cognitive outcomes showed a moderate association in one of three studies (33%) [56, 57]. Sparse literature in these areas limits definitive conclusions, though our findings highlight the need for further exploration. One study linked maternal PTSD to increased infant sleeping and eating difficulties [58], while another found reduced salivary cortisol levels in infants, suggesting possible stress dysregulation in PTSD-exposed populations [16]. Obstetric complications, such as gestational diabetes and preeclampsia, were associated with maternal PTSD in three out of five studies (60%), consistent with previous findings [59]. While evidence is growing for these associations, variability in definitions and inclusion criteria, such as excessive fetal growth and birth defects complicates interpretation [30].

### Strengths and limitations

This review aimed to comprehensively gather and assess evidence for research and clinical conclusions regarding maternal PTSD exposure and infant outcomes. To our knowledge, this is the first systematic review to incorporate both meta-analyses and a GRADE assessment, reflecting the rigor and novelty of the study. The inclusion of a comprehensive search strategy, dual reviewer screening, and data extraction processes minimized selection bias. Rigorous risk of bias (RoB) assessments and sensitivity analyses enhanced the credibility of our findings. Additionally, the use of GRADE allowed us to synthesize evidence while accounting for methodological constraints. The exploration of heterogeneity through moderator analyses further strengthened the interpretability of the results, providing a robust foundation for clinical and research applications. Several limitations must be acknowledged. Eight studies examined postpartum PTSD as an exposure, focusing on outcomes like mother-infant bonding. While aligned with the study scope, this differs from the studies on low birthweight (LBW) and preterm birth (PTB), which exclusively used prenatal PTSD as the exposure. Establishing causality remains challenging due to the observational nature of the included studies and the lack of randomized controlled trials. Many studies broadly defined “perinatal PTSD,” failing to distinguish between preexisting, incident, or persistent PTSD, limiting exploration of temporal relationships. Longitudinal designs assessing PTSD before, during, and after pregnancy are needed to clarify these dynamics. Contextual factors such as maternal stress, healthcare access, and socio-economic disparities likely influenced associations between PTSD and adverse outcomes. Systemic inequities in healthcare delivery may amplify risks for outcomes like LBW and PTB. The use of binary outcomes in meta-analyses ensured consistency but may have oversimplified nuanced relationships. Cohort studies, which constituted the majority of the evidence base, generally exhibited lower RoB but were not without limitations. Many relied on self-reported PTSD measures, introducing recall bias, and variability in assessment tools contributed to inconsistencies. While validated tools such as the PTSD Checklist (PCL) and Posttraumatic Diagnostic Scale (PDS) were frequently used [60] others used less robust measures, such as the Composite International Diagnostic Interview, which is prone to false negatives [20, 61]. Outcome reporting also varied, particularly for mother-infant bonding, limiting the ability to conduct meta-analyses for certain outcomes. Inconsistencies in birthweight and gestational age assessment methods may have introduced errors, while self-reported outcomes were subject to recall and social desirability bias, affecting reliability [62]. Sparse research on certain topics necessitated the inclusion of studies with small sample sizes, contributing to imprecision in summary estimates [63]. Wide confidence intervals observed in some analyses further highlight the need for better evidence.

### Implications and future research

Future research should prioritize longitudinal designs to explore temporal relationships and assess PTSD across multiple time points. Studies examining biomarkers of stress and interventions to mitigate PTSD’s impact on pregnancy and neonatal outcomes are warranted to advance the field further. Future research should also standardize definitions to enhance comparability across studies.

## Conclusion

The review indicates maternal PTSD links to adverse infant outcomes like smaller head circumference, eating/sleep issues, reduced breastfeeding, and lower infant cortisol. Mixed evidence exists for LBW/PTB links, though meta-analyses suggest a connection. Associations were seen for shorter GA, reduced mother-infant interaction, negative infant traits, impaired cognition, and obstetric problems due to PTSD. Limited data hint at infant cortisol and sleep/eating issues. Further studies are needed for confirmation. Given the ethical and practical constraints of conducting randomized controlled trials (RCTs) in this area, future research should focus on longitudinal cohort studies and innovative observational designs. Additionally, studies exploring biomarkers of stress and interventions for mitigating PTSD’s impact on pregnancy outcomes are warranted. Overall, this review improves understanding of how maternal PTSD affects birth, suggesting benefits in screening/treatment for PTSD during pregnancy for better outcomes.

## Supplementary Information

Below is the link to the electronic supplementary material.

Supplementary Material 1

## Data Availability

Data is provided within the manuscript or supplementary information file.

## Abbreviations

PTSD: Post-Traumatic Stress Disorder
LBW: Low Birthweight
PTB: Preterm Birth
GA: Gestational Age
SGA: Small for Gestational Age
IUGR: Intrauterine Growth Restriction
PRISMA: Preferred Reporting Items for Systematic Reviews and Meta-Analyses
GRADE: Grading of Recommendations Assessment, Development, and Evaluation
PCL: Post-traumatic Stress Disorder Checklist
DSM: Diagnostic and Statistical Manual of Mental Disorders
MINI: Mini-International Neuropsychiatric Interview
