# Supplementary material for: Associations between PTSD and pregnancy outcomes: systematic review and Meta- analysis
Source: BMC Pregnancy Childbirth. 2025 Aug 1;25:802. doi: 10.1186/s12884-025-07545-9 (PMC12317543; doi:10.1186/s12884-025-07545-9)
Supplement: Supplementary file 1 — Supplementary Material 1 [file 12884_2025_7545_MOESM1_ESM.docx]

## Additional Information

Supplementary information for this paper provided in **Appendix A**: PRISMA Checklist**, Appendix B**: Search Strategy**, Appendix C** : Main Findings for Subcategories and **Appendix E**: Codes for Analyses

#

# Appendix A: PRISMA Checklist

| **Section and Topic** | **Item #** | **Checklist item** | **Location where item is reported** |
| --- | --- | --- | --- |
| **TITLE** | | |  |
| Title | 1 | Identify the report as a systematic review. | 26 |
| **ABSTRACT** | | |  |
| Abstract | 2 | See the PRISMA 2020 for Abstracts checklist. | 26 |
| **INTRODUCTION** | | |  |
| Rationale | 3 | Describe the rationale for the review in the context of existing knowledge. | 27-28 |
| Objectives | 4 | Provide an explicit statement of the objective(s) or question(s) the review addresses. | 27-28 |
| **METHODS** | | |  |
| Eligibility criteria | 5 | Specify the inclusion and exclusion criteria for the review and how studies were grouped for the syntheses. | 29 |
| Information sources | 6 | Specify all databases, registers, websites, organisations, reference lists and other sources searched or consulted to identify studies. Specify the date when each source was last searched or consulted. | 28-29 |
| Search strategy | 7 | Present the full search strategies for all databases, registers and websites, including any filters and limits used. | 28-29 |
| Selection process | 8 | Specify the methods used to decide whether a study met the inclusion criteria of the review, including how many reviewers screened each record and each report retrieved, whether they worked independently, and if applicable, details of automation tools used in the process. | 28-29 |
| Data collection process | 9 | Specify the methods used to collect data from reports, including how many reviewers collected data from each report, whether they worked independently, any processes for obtaining or confirming data from study investigators, and if applicable, details of automation tools used in the process. | 28-29 |
| Data items | 10a | List and define all outcomes for which data were sought. Specify whether all results that were compatible with each outcome domain in each study were sought (e.g. for all measures, time points, analyses), and if not, the methods used to decide which results to collect. | 28-29 |
|  | 10b | List and define all other variables for which data were sought (e.g. participant and intervention characteristics, funding sources). Describe any assumptions made about any missing or unclear information. | 28-29 |
| Study risk of bias assessment | 11 | Specify the methods used to assess risk of bias in the included studies, including details of the tool(s) used, how many reviewers assessed each study and whether they worked independently, and if applicable, details of automation tools used in the process. | 29-30 |
| Effect measures | 12 | Specify for each outcome the effect measure(s) (e.g. risk ratio, mean difference) used in the synthesis or presentation of results. | 30 |
| Synthesis methods | 13a | Describe the processes used to decide which studies were eligible for each synthesis (e.g. tabulating the study intervention characteristics and comparing against the planned groups for each synthesis (item #5)). | 30 |
|  | 13b | Describe any methods required to prepare the data for presentation or synthesis, such as handling of missing summary statistics, or data conversions. | NA |
|  | 13c | Describe any methods used to tabulate or visually display results of individual studies and syntheses. | 30 |
|  | 13d | Describe any methods used to synthesize results and provide a rationale for the choice(s). If meta-analysis was performed, describe the model(s), method(s) to identify the presence and extent of statistical heterogeneity, and software package(s) used. | 30 |
|  | 13e | Describe any methods used to explore possible causes of heterogeneity among study results (e.g. subgroup analysis, meta-regression). | 30 |
|  | 13f | Describe any sensitivity analyses conducted to assess robustness of the synthesized results. | 30 |
| Reporting bias assessment | 14 | Describe any methods used to assess risk of bias due to missing results in a synthesis (arising from reporting biases). | 29-30 |
| Certainty assessment | 15 | Describe any methods used to assess certainty (or confidence) in the body of evidence for an outcome. | 30 |
| **RESULTS** | | |  |
| Study selection | 16a | Describe the results of the search and selection process, from the number of records identified in the search to the number of studies included in the review, ideally using a flow diagram. | 31-34 |
|  | 16b | Cite studies that might appear to meet the inclusion criteria, but which were excluded, and explain why they were excluded. | 31-34 |
| Study characteristics | 17 | Cite each included study and present its characteristics. | 31-34 |
| Risk of bias in studies | 18 | Present assessments of risk of bias for each included study. | 34-35 |
| Results of individual studies | 19 | For all outcomes, present, for each study: (a) summary statistics for each group (where appropriate) and (b) an effect estimate and its precision (e.g. confidence/credible interval), ideally using structured tables or plots. | 31-35 |
| Results of syntheses | 20a | For each synthesis, briefly summarise the characteristics and risk of bias among contributing studies. | 34-35 |
|  | 20b | Present results of all statistical syntheses conducted. If meta-analysis was done, present for each the summary estimate and its precision (e.g. confidence/credible interval) and measures of statistical heterogeneity. If comparing groups, describe the direction of the effect. | 35 |
|  | 20c | Present results of all investigations of possible causes of heterogeneity among study results. | 35 |
|  | 20d | Present results of all sensitivity analyses conducted to assess the robustness of the synthesized results. | 35 |
| Reporting biases | 21 | Present assessments of risk of bias due to missing results (arising from reporting biases) for each synthesis assessed. | 34-35 |
| Certainty of evidence | 22 | Present assessments of certainty (or confidence) in the body of evidence for each outcome assessed. | 35-56 |
| **DISCUSSION** | | |  |
| Discussion | 23a | Provide a general interpretation of the results in the context of other evidence. | 36-41 |
|  | 23b | Discuss any limitations of the evidence included in the review. | 41-42 |
|  | 23c | Discuss any limitations of the review processes used. | 41-42 |
|  | 23d | Discuss implications of the results for practice, policy, and future research. | 42-43 |
| **OTHER INFORMATION** | | |  |
| Registration and protocol | 24a | Provide registration information for the review, including register name and registration number, or state that the review was not registered. | 28 |
|  | 24b | Indicate where the review protocol can be accessed, or state that a protocol was not prepared. | 28 |
|  | 24c | Describe and explain any amendments to information provided at registration or in the protocol. | NA |
| Support | 25 | Describe sources of financial or non-financial support for the review, and the role of the funders or sponsors in the review. | NA |
| Competing interests | 26 | Declare any competing interests of review authors. | NA |
| Availability of data, code and other materials | 27 | Report which of the following are publicly available and where they can be found: template data collection forms; data extracted from included studies; data used for all analyses; analytic code; any other materials used in the review. | 69 |

Table A1. PRISMA Checklist

#

# Appendix B: Search Strategy

|  | Database | | |
| --- | --- | --- | --- |
| Concept | PubMed | GOOGLE SCHOLAR | EMBASE |
| Exposure | "stress disorders, post traumatic"[MeSH Terms] OR ("stress"[All Fields] AND "disorders"[All Fields] AND "post traumatic"[All Fields]) OR "post-traumatic stress disorders"[All Fields] OR "ptsd"[All Fields] OR ("stress disorders, post traumatic"[MeSH Terms] OR ("stress"[All Fields] AND "disorders"[All Fields] AND "post traumatic"[All Fields]) OR "post-traumatic stress disorders"[All Fields] OR ("post"[All Fields] AND "traumatic"[All Fields] AND "stress"[All Fields] AND "disorder"[All Fields]) OR "post traumatic stress disorder"[All Fields]) OR ("injuries"[MeSH Subheading] OR "injuries"[All Fields] OR "trauma"[All Fields] OR "wounds and injuries"[MeSH Terms] OR ("wounds"[All Fields] AND "injuries"[All Fields]) OR "wounds and injuries"[All Fields] OR "trauma s"[All Fields] OR "traumas"[All Fields]) | PTSD, OR trauma, OR posttraumatic stress disorder OR traumatic experience OR traumatic exposure OR trauma victim OR stress disorder OR severe trauma OR trauma disorder | (PTSD or posttraumatic stress disorder or trauma or traumatic exposure or traumatic experience or severe trauma or stress disorder or traumas or severe stressor).af. |
| Population | "pregnant women"[MeSH Terms] OR ("pregnant"[All Fields] AND "women"[All Fields]) OR "pregnant women"[All Fields] OR ("pregnant"[All Fields] OR "pregnants"[All Fields]) OR ("pregnancy"[MeSH Terms] OR "pregnancy"[All Fields] OR "pregnancies"[All Fields] OR "pregnancy s"[All Fields]) OR ("gestate"[All Fields] OR "gestated"[All Fields] OR "gestates"[All Fields] OR "gestating"[All Fields] OR "gestational"[All Fields] OR "gestations"[All Fields] OR "pregnancy"[MeSH Terms] OR "pregnancy"[All Fields] OR "gestation"[All Fields]) OR ("maternally"[All Fields] OR "maternities"[All Fields] OR "maternity"[All Fields] OR "mothers"[MeSH Terms] OR "mothers"[All Fields] OR "maternal"[All Fields]) | pregnant OR pregnancy, OR gestation, OR maternal, OR pregnant women OR pregnant persons OR expecting mother OR gestation | (Pregnancy or pregnant women or gestation or obstetrics or maternal or expectant mother or pregnant persons or pregnant).af. |
| Outcome | “pregnancy outcome"[MeSH Terms] OR ("pregnancy"[All Fields] AND "outcome"[All Fields]) OR "pregnancy outcome"[All Fields] OR (("birth s"[All Fields] OR "birthed"[All Fields] OR "birthing"[All Fields] OR "parturition"[MeSH Terms] OR "parturition"[All Fields] OR "birth"[All Fields] OR "births"[All Fields]) AND ("outcome"[All Fields] OR "outcomes"[All Fields])) OR (("fetale"[All Fields] OR "fetally"[All Fields] OR "fetals"[All Fields] OR "fetus"[MeSH Terms] OR "fetus"[All Fields] OR "fetal"[All Fields] OR "foetal"[All Fields]) AND ("outcome"[All Fields] OR "outcomes"[All Fields])) OR (("obstetric"[All Fields] OR "obstetrically"[All Fields] OR "obstetrics"[MeSH Terms] OR "obstetrics"[All Fields] OR "obstetrical"[All Fields]) AND ("outcome"[All Fields] OR "outcomes"[All Fields])) | pregnancy outcome OR birth outcome, OR fetal outcome OR baby outcome OR pregnancy complications OR adverse birth outcome OR obstetric outcomes OR birth complications OR neonatal outcomes | (pregnant outcomes or pregnancy outcome or birth outcomes or neonatal outcomes or obstetric outcome or pregnancy complications or fetal outcomes or adverse birth outcomes or obstetrical complications).af.) |
| Linking concepts | 1 AND 2 AND 3  N=200 | 1 AND 2 AND 3  N=168 | 1 AND 2 AND 3  N=42 |

#

# Appendix C : Main Findings for Subcategories

| Study | Main Findings |
| --- | --- |
| Blackmore et al. (2016) | **Of the 358 deliveries, 29 (8.1%) were considered low birthweight (< 2500g) Univariate analysis showed that birthweight was not significantly associated with either history (r = −.10, p = .850) or frequency of traumatic events (r = .00, p = .936),** |
| Engel et al. (2005) | **Probable PTSD was not associated with birthweight** |
| Ferri et al. (2007)* | **PTSD was associated with low birth weight,  after adjusting for confounders and mediator PR = 1.91* (95%CI 1.01–3.63)** |
| Gelaye et al. (2020) | **PTSD was not associated with low birth weight (LBW) at delivery.** |
| Koen et al. (2016) | **No association was observed between maternal diagnosed  life-long PTSD and decreased standard deviation scores of weight-for-age  (WAZ score)** |
| Lipkind et al. (2010)* | **Probable PTSD was significantly associated with a difference in birth weight (unadjusted), but this was not significant when controlling for confounding variables. Low birth weight was two-times more likely in women with high PTSD scores.** |
| Maslow et al. (2016)* | **Probable 9/11-related posttraumatic stress disorder 2 to 3 years after 9/11 were associated with low  birth weight (LBW) during the early study period.** |
| Morland et al. (2007) | **PTSD was not significantly associated with low birthweight** |
| Rashind et al. (2020)* | **In univariate analysis model, PTSD was significantly associated with low birthweight (LBW). In logistic regression model, PTSD was independently associated with low birthweight (LBW) in the presence of other factors like maternal / paternal schooling, gravida, history of preterm, BMI of the mother and maternal anemia** |
| Rogal et al. (2007) | **low birth weight was not significantly associated with antenatal PTSD** |
| Rosen et al. (2007)* | **Those respondents who were experiencing both a mental health condition (one of which being PTSD) and IPV had the highest odds (2.5 time greater of having a low birth weight infant (p=0.026). The odds of having a low bwt baby was 2.1 timesgreater in those women who had ptsd (p= 0.017)** |
| Seng et al. (2011)* | **Current PTSD symptom count was significantly associated with lower birth weight, compared to trauma-exposed resilient cohort and non-exposed cohort; negative correlation of current PTSD symptom count with birthweight was significant (P<0.001)** |
| Weinreb et al. (2018) | **There were no significant differences between low birthtweight oucome for partipants who received the intervention for PTSD compared to those  who did not (χ2 = 3.62, df = 3, p = .306)** |
| Xiong et al. (2008) | **The frequency of low birth weight was higher in women with PTSD (23.1%) and with depression (11.6%)  than that in women without PTSD (9.1%) Antenatal PTSD found to be associated with increased risk of low birth weight – low birth weight was three-times more likely in women with antenatal PTSD.** |
| Feeley et al. (2011)* | **Mothers who reported more PTSD symptoms had infants who weighed less at birth** |
| * is significant |  |

Table C1: Main Findings for Low Birthweight (n=15)

| Study | Main Findings |
| --- | --- |
| Blackmore et al. (2016) | **trauma history (r = .05, p = .336) and frequency of traumatic events (r = .04, p = .430) were not significantly associated with gestational age** |
| Engel et al. (2005) | **Probable PTSD was not associated with gestational duration** |
| Gelaye et al. (2020) | **PTSD was not associated with gestational age at delivery.** |
| Harville et al. (2015)* | **For PTSD, the associations were in the direction of PTSD being associated with reduced gestational age (adjusted beta -2.85 days, p = 0.17)** |
| Koen et al. (2016) | **No association was observed between maternal diagnosed life-long PTSD and  small for gestational age (SGA)** |
| Lipkind et al. (2010) | **Probable PTSD was not associated with a difference in gestational age of delivery.** |
| Lutgendorf et al. (2021) | **compared to service members without PTSD.PTSD case status was not associated with size for gestational age (SGA).** |
| Rogal et al. (2007) | **gestational age was not significantly associated with antenatal PTSD** |
| Weinreb et al. (2018) | **There were no significant differences in gestational age for partipants who received the intervention for PTSD  compared to those who did not** |
| * is significant |  |

Table C2. Main Findings for Shorter GA (n=9)

| Study | Main Findings |
| --- | --- |
| Gelaye et al. (2020)* | **Compared to those without PTSD, women with PTSD (34.5%)  had higher odds of delivering preterm (OR = 1.28; 95%CI: 1.00–1.65)** |
| Harville et al. (2015)* | **For PTSD, the associations were in the direction of  PTSD being associated with higher preterm birth rate  (adjusted OR 3.61, 0.93–14.03)** |
| Haviland et al. (2021) | **Compared to participants with less perceived stress, the risk of preterm delivery was no different among participants with a moderate score perceived stress (RR 1.23, 95% CI 0.68, 2.25) and a high score of perceived stress (RR 1.62, 95% CI 0.73, 3.62)** |
| Koen et al. (2016)* | **No association was observed between maternal diagnosed life-long PTSD and preterm delivery** |
| Lipkind et al. (2010)* | **Preterm delivery was two-times more likely in women with high PTSD scores.** |
| Lutgendorf et al. (2021) | **Compared to service members without PTSD.  PTSD case status was not associated with preterm birth,** |
| MacGinty et al. (2020) | **No association was observed between antenatal maternal  psychological distress and preterm birth** |
| Morland et al. (2007) | **PTSD was not significantly associated with  preterm birth (pre-term contractions)** |
| Rogal et al. (2007) | **Preterm delivery was not significantly associated with antenatal PTSD. However, an association was observed in that preterm delivery was nearly three-times more likely in mothers with antenatal PTSD (although not statistically significant)** |
| Seng et al. (2011) | **Current PTSD was not significantly associated with pre-term birth.** |
| Weinreb et al. (2018) | **There were no significant differences between preterm delivery outcomes for those who received the intervention for PTSD compared to those who did not (χ2 = 2.203, df = 3, p = .531)** |
| Xiong et al. (2008) | **Antenatal PTSD was associated with decreased risk of preterm birth** |
| Yonkers et al. (2014) * | **Risk of preterm birth was elevated in women with a likely diagnosis of PTSD (Adjusted OR = 1.22, 95% C.I. 0.57–2.61)** |
| Shaw et al. (2014)* | **Spontaneous preterm delivery was higher in those  with active PTSD (9.2%, n=5176) than those with historical (8.0%, n=590) or no PTSD (7.4%, n=5982) before adjustment (P= .02).** |
| * is significant |  |

Table C3. Main Findings for Preterm Birth (n=14)

| Study | Main Findings |
| --- | --- |
| Feeley et al. (2011)* | **Mothers who reported more PTSD symptoms were less sensitive and less effective at structuring interactions with their infant.** |
| Parfitt et al. (2013) | **Maternal PTSD was not significantly correlated with any maternal sensitivity, control, or unresponsiveness** |
| Parfitt & Ayers (2009)* | **PTSD was significantly correlated with mother-infant interaction. Mothers with PTSD reported a significantly poorer relationship with their infant.** |
| Muller-Nix et al. (2004)* | **At 6 months: high-stress post partum PTSD  mothers of preterm infants were associated with significantly lower maternal sensitivity and significantly higher maternal control compared with full-term mothers At 18 months: high-stress post partum  PTSD mothers of preterm infants were associated with significantly greater infant compliance and passivity** |
| Ayers et al. (2007) | **Maternal PTSD symptoms were not associated with the mother-baby bond.** |
| Davies et al. (2008)* | **Mothers with FT or PS PTSD symptoms perceived their attachment to be significantly less to their infants, Mothers with FT or PS PTSD also perceived greater infant-directed hostility and reduced  pleasure when interacting with their infants. FT mothers also reported that they had significantly less desire in being within close proximity to their infant** |
| Seng et al. (2013) * | **Pre-existing maternal PTSD was an elevated risk factor for impaired bonding.** |
| Ionio et al. (2014) * | **Infants whose mothers had higher PTSD symptoms at two months physically distanced themselves from their mother. Data showed that persistence of PTSD symptoms had a different effect on early mother–child interactions than those of mothers who have not had postpartum stress symptoms** |
| Parfit et al. (2014) | **Maternal PTSD at three months postpartum was not significantly correlated with a poorer baby-bond at 3- months and 15-months postpartum.** |
| Mcdonald et al. (2011) | **PTSD babies showed greater amounts of hard crying when reuniting with their mothers than during the baseline play episode Infants of mothers without elevated symptoms of PTSD showed higher amounts of fussing in the second reunion with their mothers relative to the first reunion but low levels of hard crying throughout the procedure. Early PTSD symptoms (at either 6 weeks or 3 months postpartum) was not significantly correlated with maternal perception of the child at 2 years postpartum.** |
| Breastfeeding |  |
| Beck et al. (2011)* | **Women with higher PTSD scores were significantly less likely to have breastfed their infant for as long as they wanted to, and were significantly less likely to be exclusively breast-feeding at 1 month postpartum.** |
| Halperin et al. (2015)* | **Significantly more women with PTSD symptoms did not breastfeed their infant.** |
| * is significant |  |

Table C4.Main Findings for Reduced Mother-Infant Interaction (n=12)

| Study | Main Findings |
| --- | --- |
| Infant NA |  |
| Campbell et al. (2020)* | **significant positive linear association between the prenatal stress and infant negative affectivity (NA). For each one-unit increase in prenatal stress  index, the infant NA score increased by 0.40 (0.16–0.64) (b [95% confidence interval], and PTSD was the strongest contributor to the prenatal stress score in Hispanic women** |
| Parfitt et al. (2013) | **Maternal PTSD was not significantly correlated with infant cooperation, difficulty, compliance or passivity** |
| Infant temperament |  |
| Bosquet Enlow et al. (2011) | **Maternal PTSD symptoms were not significantly associated with measures of infant emotional reactivity. Maternal PTSD symptoms were significantly correlated with maternal reports of the infant's ability to recover once distressed. Maternal PTSD showed minimal associations with infant emotional reactivity although infants of mothers with symptoms of PTSD to show a greater increase in hard crying during the second still-face episode relative to the first still-face episode. Maternal PTSD was associated with infant recovery from distress; infants of mothers with symptoms of PTSD showed greater amounts of hard crying during the second reunion than during the baseline play episode, the first reunion, and the second still-face episodes. Infants of mothers without elevated symptoms of PTSD showed higher amounts of fussing in the second reunion relative to the first reunion but low levels of hard crying throughout the procedure.** |
| Davies et al. (2008)* | **Mothers with FT or PS PTSD symptoms perceived their infants to be more difficult in temperament.** |
| Cognition |  |
| MacGinty et al. (2020) | **No association was observed between antenatal maternal psychological  distress and early developmental outcomes** |
| Feeley et al. (2011) | **Maternal PTSD symptom score was not related to infant cognitive development at 6-months postpartum.** |
| Parfitt et al. (2014a)* | **Maternal postpartum PTSD was moderately associated with poorer cognitive outcomes, but was not significantly associated with language or motor scores.** |
| Sleeping/Eating Behaviour |  |
| Pierrehumbert et al. (2003)* | **There was a statistically significant difference between the aggregated index of problems (sleeping and eating), with significantly more difficulties with premature infants of mothers with PTSD, with sleeping problems being most affected.** |
| Cortisol |  |
| Yehuda et al. (2005)* | **Infant salivary cortisol was lower in infants  of women with PTSD. Lower cortisol levels were most apparent in babies born to mothers with PTSD in their hird trimesters on 9/11** |
| * is significant |  |

Table C5. Main Findings for Infant & Neonatal Complications (n=9)

| Study | Main Findings |
| --- | --- |
| Engel et al. (2005)* | **PTSS (Post Traumatic Stress Symptomology) was inversely associated with infant head circumference at birth, such that a 1-unit increase in PCL score was associated with a 0.07 cm decrement in head circumference (P = 0.01)** |
| Koen et al. (2016)* | **Maternal trauma was  significantly associated with a 0.3 unit reduction in infant HCAZ (head circumference) scores at birth  (95% CI: 0.1; 0.5) This association remained significant when adjusted for study site, SES, and recent life stressor** |
| MacGinty et al. (2020)* | **antenatal maternal psychological distress  was associated with a smaller  head circumference at birth  (coefficient=−0.30, 95% CI: −0.49; −0.10).** |
| * is significant |  |

Table C6. Main Findings for Reduced Head Circumference (n=3)

| Study | Main Findings |
| --- | --- |
| Blackmore et al. (2016) | **Neither trauma history, χ2 (1, N = 358) = 0.51, p = .473; nor frequency of traumas, χ2 (3, N = 358) = 3.49, p = .323, was significantly associated with obstetric complication** |
| Lutgendorf et al. (2021) | **compared to service members without PTSD.  PTSD case status was not associated  with major birth defects RR 1.03,( 95% CI 0.79–1.34)** |
| Nillni et al. (2020)* | **PTSD symptoms, aOR = 1.16, 95% CI [1.00, 1.35],  significantly predicted an increased  risk of an adverse pregnancy outcome** |
| Seng et al. (2001)* | **Logistical regression model found five obstetric complications to be significantly associated with maternal PTSD, one of which was excessive fetal growth** |
| Shaw et al. (2017)* | **current PTSD diagnosis (reference = no PTSD) was associated with an increased risk of GDM (RR 1.4, 95% confidence interval (CI) 1.2, 1.7) and preeclampsia (RR 1.3, 95% CI 1.1, 1.6). PTSD also predicted prolonged (>4 day) delivery hospitalization (RR 1.2, 95% CI 1.01, 1.4), and repeat hospitalizations (RR 1.4, 95% CI 1.2, 1.6), but not caesarean delivery.** |
| * is significant |  |

Table C7.Main Findings for Obstetric Complications (n=5)

| Study | Main Findings |
| --- | --- |
| Head Circumference |  |
| Engel et al. (2005)* | **PTSS (Post Traumatic Stress Symptomology) was inversely associated with infant head circumference at birth, such that a 1-unit increase in PCL score was associated with a 0.07 cm decrement in head circumference (P = 0.01)** |
| Koen et al. (2016)* | **Maternal trauma was significantly associated  with a 0.3 unit reduction in infant HCAZ (head circumference) scores at birth (95% CI: 0.1; 0.5) This association remained significant when adjusted for study site, SES, and recent life stressor** |
| MacGinty et al. (2020)* | **Antenatal maternal psychological distress  was associated with a smaller  head circumference at birth  (coefficient=−0.30, 95% CI: −0.49; −0.10).** |
| Breastfeeding |  |
| Beck et al. (2011)* | **Women with higher PTSD scores were  significantly less likely to have breastfed their infant for as long as they wanted to, and were significantly less likely to be  exclusively breast-feeding at 1 month  postpartum.** |
| Halperin et al. (2015)* | **Significantly more women with PTSD symptoms did not breastfeed their infant.** |
| Sleeping/Eating Behaviour |  |
| Pierrehumbert et al. (2003)* | **There was no significant difference between preterm infants of mothers with high or low PPQ, and controls, in relation to sleeping or eating difficulties. However, there was a statistically significant difference between the aggregated index of problems (sleeping and eating), with significantly more difficulties with premature infants of mothers with PTSD, with sleeping problems being most affected.** |
| Cortisol |  |
| Yehuda et al. (2005)* | **Infant salivary cortisol was lower in infants  of women with PTSD. Lower cortisol levels were most apparent in babies born to mothers with PTSD in their third trimesters on 9/11** |
| * is significant |  |

Table C8.Evidence for Overall Associations

# Appendix D: Sensitivity Output

| tau^2 (estimated amount of residual heterogeneity): 0 (SE = 0.5190) |
| --- |
| tau (square root of estimated tau^2 value): 0 |
| I^2 (residual heterogeneity / unaccounted variability): 0.00% |
| H^2 (unaccounted variability / sampling variability): 1.00 |
| R^2 (amount of heterogeneity accounted for): 100.00% |
|  |
| Test for Residual Heterogeneity:  QE(df = 1) = 0.4065, p-val = 0.5237  Test of Moderators (coefficients 2:9):  QM(df = 8) = 28.0447, p-val = 0.0005 |
| estimate se zval pval ci.lb ci.ub |
|  |
| countryPakistan 3.6806 1.1943 3.0817 0.0021 1.3398 6.0214 ** |
| Case-Control 1.5444 0.7181 2.1507 0.0315 0.1369 2.9518 * |
| Prospective Cohort 1.6547 0.6294 2.6289 0.0086 0.4210 2.8884 ** |
| Signif. codes: 0 ‘***’ 0.001 ‘**’ 0.01 ‘*’ 0.05 ‘.’ 0.1 ‘ ’ 1 |

Table D1 Sensitivity Analysis for Low BWT

| **tau^2 (estimated amount of residual heterogeneity): 0 (SE = 0.0108)** |
| --- |
| **tau (square root of estimated tau^2 value): 0** |
| **I^2 (residual heterogeneity / unaccounted variability): 0.00%** |
| **H^2 (unaccounted variability / sampling variability): 1.00** |
| **R^2 (amount of heterogeneity accounted for): 0.00%** |
|  |
| **Test for Residual Heterogeneity:** |
| **QE(df = 3) = 2.4423, p-val = 0.4858** |
|  |
| **Test of Moderators (coefficients 2:6):** |
| **QM(df = 5) = 6.5415, p-val = 0.2570** |

**Table D2:** **Sensitivity Analysis for Preterm Birth**

# Appendix E: Codes for Analyses

## Section E1: Code for LBW Analysis

library(metafor)

# create the dataset

publisher<- c("Blackmore et al. (2016)", " Engel et al. (2005)", " Ferri et al. (2007) ", " Gelaye et al. (2020)" , " Koen et al. (2016)", " Maslow et al. (2016)", " Morland et al. (2020)", " Rogal et al. (2007)", " Seng et al. (2011)", " Xiong et al. (2008)", " Feeley et al. (2011)", " Rosen et al. (2007)", " Lipkind et al. (2010)", " Rashind et al. (2020)", " Weinreb et al. (2018)")

APTSDposbwtpos <- c(NA, NA, 13, 128, NA, NA, NA, 2, 13, 2, 5, NA, NA, 71, 5)

BPTSDposbwtneg <- c(NA, NA, 64, 1433, NA ,NA, NA, 29, 242, 11, 0, NA, NA, 13, 55)

CPTSDngbwtpos<- c(NA, NA, 49, 128, NA, NA, NA, 70, 24, 27, 16, NA , NA, 154, 13)

DPTSDngbwtneg<- c(NA, NA, 669, 2761, NA, NA, NA, 1009, 560, 258, 0, NA, NA, 212 ,76)

country <- c("USA", "USA", "Brazil", "Peru", "South Africa", "USA", "USA", "USA", "USA", "USA", "Canada", "USA", "USA", "Pakistan", "USA")

assessmenttool <- c("DSM", "PCL", "CIDI", "PCL", "MINI", "PCL", "PCL", "MINI",

                    "National Women's StudyPTSD Module", "PCL", "PPQ",

                    "University of Michigan Composite International Diagnostic Interview (UM-CIDI)",

                    "PCL", "MINI", "Four-item Primary Care-PTSD Screen")

studydesign <- c("Prospective Cohort", "Prospective Cohort", "Prospective Cohort",

                 "Prospective Cohort", "Prospective Cohort", "Prospective Cohort",

                 "Prospective Cohort", "Prospective Cohort", "Prospective Cohort",

                 "Prospective Cohort", "Cross-Sectional", "Retrospective Cohort",

                 "Case-Control", "Case-control", "Case-control")

OR <- c(NA, NA, NA, NA, NA, 3, NA, NA, NA, NA, NA, NA, 2.49, NA, NA)

CIlower<- c(NA, NA, NA, NA, NA, 1.6, NA, NA, NA, NA, NA, NA, 1.02, NA, NA)

CIupper<-c(NA, NA, NA, NA, NA, 5.6, NA, NA, NA, NA, NA, NA, 6.08, NA, NA)

Pvalue<- c(0.85, 0.69, NA, NA, NA, NA, NA, NA, NA, NA, NA, NA, NA, NA, NA)

 Totalsample <- c(358, 51, 795 ,4450, 366 ,3271, 101 ,1100, 839, 298, 21, 148, 446, 450, 149)

metaptsd <- data.frame(publisher, APTSDposbwtpos, BPTSDposbwtneg, CPTSDngbwtpos, DPTSDngbwtneg, Totalsample, OR, Pvalue, CIlower, CIupper, country, assessmenttool, studydesign)

#do analysis

 metaptsd

 bwtmeta1 <- escalc(measure="OR", ai= APTSDposbwtpos, bi=BPTSDposbwtneg, ci=CPTSDngbwtpos, di=DPTSDngbwtneg, sei=TransOR, data=metaptsd)

 bwtmeta1

 analysis1 <- rma(yi, vi, data=bwtmeta1)

 analysis1

 predict(analysis1, transf=exp, digits=2)

bwtmeta2 <- data.frame(summary(bwtmeta1))

bwtmeta2 <- escalc(measure="OR", ai= APTSDposbwtpos, bi=BPTSDposbwtneg, ci=CPTSDngbwtpos, di=DPTSDngbwtneg, sei=TransOR, data=bwtmeta2)

bwtmeta2  <- conv.wald(out=OR, ci.lb=CIlower, ci.ub=CIupper, pval=Pvalue, n=Totalsample, data=bwtmeta2, transf=log)

Bwtmeta2

#create random effect model meta-analysis and forest plot

analysis2 <- rma(yi, vi, data=bwtmeta2)

predict(analysis2, transf=exp, digits=2)

predict(analysis2, transf=exp, digits=2)

forest(analysis2, transf=exp, slab = paste(publisher))

#sensitivity analysis for country, assesment tool and study design

metaptsd <- data.frame(publisher, APTSDposbwtpos, BPTSDposbwtneg, CPTSDngbwtpos, DPTSDngbwtneg, Totalsample, OR, Pvalue, CIlower, CIupper, country, assessmenttool, studydesign)

 metaptsd

 bwtmeta1 <- escalc(measure="OR", ai= APTSDposbwtpos, bi=BPTSDposbwtneg, ci=CPTSDngbwtpos, di=DPTSDngbwtneg, sei=TransOR, data=metaptsd)

 bwtmeta1

 metaregmodel <- rma(yi, vi, mods = ~ country + studydesign + assessmenttool, random = ~ 1 | publisher, data = bwtmeta1)

 metaregmodel

 forest(metaregmodel, transf=exp, slab = paste(publisher), xlim = c(-50, 50))

bwtmeta2 <- data.frame(summary(bwtmeta1))

bwtmeta2 <- escalc(measure="OR", ai= APTSDposbwtpos, bi=BPTSDposbwtneg, ci=CPTSDngbwtpos, di=DPTSDngbwtneg, sei=TransOR, data=bwtmeta2)

bwtmeta2  <- conv.wald(out=OR, ci.lb=CIlower, ci.ub=CIupper, pval=Pvalue, n=Totalsample, data=bwtmeta2, transf=log)

bwtmeta2

 metaregmodel2<- rma(yi, vi, mods = ~ country + studydesign + assessmenttool, random = ~ 1 | publisher, data = bwtmeta2)

 metaregmodel2

forest(metaregmodel2, slab = paste(publisher), xlim = c(-50, 50), col = "red", addfit = TRUE, digits = 2, mlab = "Sensitivity

## Section E2: Code for PTB Analysis

#forpreterm

remotes::install_github("wviechtb/metafor")

install.packages("remotes")

force=TRUE

library(metafor)

#create dataset

  publisher<- c("Yonkers et al. (2014)",  "Shaw et al. (2014)", "Harville et al. (2015)", "Gelaye et al. (2020)", "Koen et al. (2016)",  "Haviland et al. (2021)", "Morland et al. (2020)", "Rogal et al. (2007)",  "Seng et al. (2011)", "Xiong et al. (2008)", "Lutgendorf et al. (2021)", "Lipkind et al. (2010)", "MacGinty et al. (2020)", "Weinreb et al. (2018)")

 APTSDpospretermpos <- c(13, 175, 4, 112, NA, NA,NA, 5, NA, 1, 141, NA, NA, 3)

  BPTSDpospretermneg <- c(3, 1746, 22, 1407, NA, NA, NA, 26, NA, 12, 1516, NA, NA, 57)

 CPTSDngpretermpos<- c(114, 982, 15, 167, NA, NA, NA, 76, NA, 28, 7817, NA, NA, 8)

  DPTSDngpretermneg<- c(44, 12303, 248, 2722, NA, NA, NA, 1003, NA, 257, 93747, NA, NA, 81)

 Totalsample <- c(174, 15206, 289, 4408 ,NA, NA, 101, 1110, NA, 298, 103221, NA, 961,149)

 OR <- c(1.22,NA, 3.61,1.28,2.3,NA,NA ,NA ,NA ,0.8 ,1.1, 2.67 ,NA,NA)

CIlower<- c(0.57 ,NA, 0.93,1 ,0.82 ,NA, NA, NA, NA,0.1, NA, NA, NA, NA)

CIupper<-c(2.61, NA, 14.03, 1.65 ,6.38 ,NA, NA ,NA, NA, 6.39, NA, 1.23, NA, NA)

study_design <- c("Prospective Cohort", "Retrospective Cohort", "Prospective Cohort", "Prospective Cohort", "Prospective Cohort", "Prospective Cohort", "Prospective Cohort", "Prospective Cohort", "Prospective Cohort", "Prospective Cohort", "Retrospective Cohort", "Case Control", "Prospective Cohort", "Case Control")

country <- c("USA", "USA", "USA", "Peru", "South Africa", "USA", "USA", "USA", "USA", "USA", "USA", "USA", "South Africa", "USA")

assessment_tool <- c("Antenatal PTSD MPSS", "Antenatal PTSD MPSS", "PCL", "PCL", "MINI", "Cohen's 4-item Perceived Stress Scale", "PCL", "MINI", "National Women's Study PTSD Module", "PCL", "Antenatal PTSD MPSS", "PCL", "(SRQ-20)", "Four-item Primary Care-PTSD Screen")

Pvalue<- c(NA,0.2,0.06,NA, NA,NA,NA,NA,0.067, NA, NA ,NA ,NA, NA)

metaptsd <- data.frame(publisher, APTSDpospretermpos,  BPTSDpospretermneg, CPTSDngpretermpos, DPTSDngpretermneg, Totalsample, OR, Pvalue, CIlower, CIupper)

#conduct analysis

metaptsd

 pretermmeta1 <- escalc(measure="OR", ai= APTSDpospretermpos, bi=BPTSDpospretermneg, ci=CPTSDngpretermpos, di=DPTSDngpretermneg, sei=TransOR, data=metaptsd)

  pretermmeta1

  analysis1 <- rma(yi, vi, data=pretermmeta1)

 analysis1

 predict(analysis1, transf=exp, digits=2)

 analysis1 <- rma(yi, vi, data=pretermmeta1)

 analysis1

 predict(analysis1, transf=exp, digits=2)

 pretermmeta2 <- data.frame(summary(pretermmeta1))

pretermmeta2<- escalc(measure="OR", ai= APTSDpospretermpos, bi=BPTSDpospretermneg, ci=CPTSDngpretermpos, di=DPTSDngpretermneg, data=metaptsd)

pretermmeta2  <- conv.wald(out=OR, ci.lb=CIlower, ci.ub=CIupper, pval=Pvalue, n=Totalsample, data=pretermmeta2, transf=log)

pretermmeta2

#random effect and forest plot

analysis2 <- rma(yi, vi, data=pretermmeta2)

predict(analysis2, transf=exp, digits=2)

predict(analysis2, transf=exp, digits=2)

forest(analysis2, transf=exp, slab = paste(publisher))

# conduct sensitivity mods adjustment

metaptsd <- data.frame(publisher, APTSDpospretermpos,  BPTSDpospretermneg, CPTSDngpretermpos, DPTSDngpretermneg, Totalsample, OR, Pvalue, CIlower, CIupper, country, assessment_tool, study_design)

 metaptsd

  pretermmeta1 <- escalc(measure="OR", ai= APTSDpospretermpos, bi=BPTSDpospretermneg, ci=CPTSDngpretermpos, di=DPTSDngpretermneg, sei=TransOR, data=metaptsd)

  pretermmeta1

 metaregmodel <- rma(yi, vi, mods = ~ country + study_design + assessment_tool, random = ~ 1 | publisher, data =  pretermmeta1)

 metaregmodel

 forest(metaregmodel, transf=exp, slab = paste(publisher), xlim = c(-50, 50))

pretermmeta2 <- data.frame(summary(pretermmeta1))

pretermmeta2 <- escalc(measure="OR", ai= APTSDpospretermpos, bi=BPTSDpospretermneg, ci=CPTSDngpretermpos, di=DPTSDngpretermneg, sei=TransOR, data=pretermmeta2)

pretermmeta2 <- conv.wald(out=OR, ci.lb=CIlower, ci.ub=CIupper, n=Totalsample, data=pretermmeta2, transf=log)

pretermmeta2

 metaregmodel2<- rma(yi, vi, mods = ~ country + study_design + assessment_tool, random = ~ 1 | publisher, data = pretermmeta2)

 metaregmodel2

 forest(metaregmodel2, slab = paste(publisher), xlim = c(-50, 50), col = "red", addfit = TRUE, digits = 2, mlab = "Sensitivity analysis")

# 
